# Application of Bimetallic Hydroxide/Graphene Composites in Wastewater Treatment

**DOI:** 10.3390/molecules29133157

**Published:** 2024-07-02

**Authors:** Dan Chen, Jiao Wang, Nana Li, Xiaoqin Luo, Hua Yu, Haichang Fu, Zhangxin Chen, Binbin Yu, Yanxian Jin, Dmitry S. Kopchuk

**Affiliations:** 1 School of Pharmaceutical and Chemical Engineering, Taizhou University, Taizhou 318000, China; chendan824@126.com (D.C.); chunmianhua0525@163.com (J.W.); linana1640@163.com (N.L.); lxq18886201542@163.com (X.L.); yh624304@163.com (H.Y.); hcfu@tzc.edu.cn (H.F.); 2 Taizhou Biomedical and Chemistry Industry Institute, Taizhou 318000, China; 3 Chemical Engineering Institute, Ural Federal University, 19 Mira Str., 620002 Ekaterinburg, Russia; dkopchuk@mail.ru

**Keywords:** antibiotic wastewater, electro-Fenton technology, LDHs/rGO, degradation

## Abstract

The increasing discharge of antibiotic wastewater leads to increasing water pollution. Most of these antibiotic wastewaters are persistent, strongly carcinogenic, easy to bioaccumulate, and have other similar characteristics, seriously jeopardizing human health and the ecological environment. As a commonly used wastewater treatment technology, non-homogeneous electro-Fenton technology avoids the hazards of H_2_O_2_ storage and transportation as well as the loss of desorption and reabsorption. It also facilitates electron transfer on the electrodes and the reduction of Fe^3+^ on the catalysts, thereby reducing sludge production. However, the low selectivity and poor activity of electro-synthesized H_2_O_2_, along with the low concentration of its products, combined with the insufficient activity of electrically activated H_2_O_2_, results in a low ∙OH yield. To address the above problems, composites of layered bimetallic hydroxides and carbon materials were designed and prepared in this paper to enhance the performance of electro-synthesized H_2_O_2_ and non-homogeneous electro-Fenton by changing the composite mode of the materials. Three composites, NiFe layered double hydroxides (LDHs)/reduced graphene oxide (rGO), NiMn LDHs/rGO, and NiMnFe LDHs/rGO, were constructed by the electrostatic self-assembly of exfoliated LDHs with few-layer graphene. The LDHs/rGO was loaded on carbon mats to construct the electro-Fenton cathode materials, and the non-homogeneous electro-Fenton oxidative degradation of organic pollutants was realized by the in situ electrocatalytic reduction of O_2_ to ∙OH. Meanwhile, the effects of solution pH, applied voltage, and initial concentration on the performance of non-homogeneous electro-Fenton were investigated with ceftazidime as the target pollutant, which proved that the cathode materials have an excellent electro-Fenton degradation effect.

## 1. Introduction

Wastewater emissions, particularly those from industrial sources, continue to rise, leading to increasing levels of water pollution [1,2]. Industrial wastewater is highly complex, containing toxic organic matter that is difficult to biodegrade [1]. Many of these organic compounds are persistent, strongly carcinogenic, and prone to bioaccumulation, among other characteristics. They can be amplified through the food chain step by step and persist in the environment for long periods, undergoing long-distance migration, thereby posing a serious threat to human health and the ecological environment [3]. Antibiotic wastewater is one of the more challenging types of industrial wastewater to treat [4,5].

The difficulty in treating antibiotic wastewater can be attributed to two reasons. First, antibiotics enter the environment with production wastewater and waste residue during the production process, bringing about environmental pollution problems that cannot be ignored [3,6]. Secondly, about 48% of antibiotics are used in the treatment of human diseases, and the rest are used in animal husbandry [3,7,8]. However, due to the limitations of the organism’s own metabolism, the absorption and utilization of antibiotics by human beings or animals is very limited, and about 30–90% of antibiotics will be excreted in the form of original drugs or metabolites with the feces or urine of the organisms and enter the ecological environment after use [9]. These will not only threaten human health, but also bring irreversible and serious damage to the ecological environment on which humans depend [9]. Low concentrations of antibiotics and their metabolites in water can induce the generation of resistance genes, potentially exerting toxic effects on aquatic organisms and humans. Pathogens can develop resistance to antibiotic treatment under such conditions [10]. Antibiotic-resistant pathogens caused more than 50,000 deaths in Europe and the United States in 2015, and the number of deaths is projected to rise to 10 million per year globally by 2050 [11]. The World Health Organization has identified antibiotic drug resistance as a global public health crisis and has proposed that it must be urgently addressed [12]. Therefore, the effective management of antibiotic wastewater is of great significance to the sustainable and coordinated development of human social environment and ecosystem.

The traditional methods of wastewater treatment are physical, biological, and chemical [3]. However, most of these traditional treatment methods are limited by the instability of microbial flora, long degradation periods, large dosage, sludge, and hazardous waste generation, as well as treatment costs [2]. Therefore, it is of great practical significance to develop highly efficient, stable, environmentally friendly, and sustainable wastewater treatment technologies. Electrochemical advanced oxidation technology, compared to traditional water treatment technology, has unique advantages: (1) the reaction process is clean, with electrons as reactants, almost no addition of chemical reagents, and no secondary pollution [13,14]; (2) it is controllable, with both the reaction rate and the direction of the reaction easily controlled by changing the current or potential [13,14]; (3) it has multifunctionality and can be realized with advanced oxidation technology to achieve the coupling effect, for example, the combination of electrochemical oxygen reduction technology with the Fenton technology electrically activates the sulfate technology, etc. [13,15]; and (4) it has a high degree of automation, so the reaction can be carried out at room temperature and a normal pressure, the electrodes and the reactor are flexible, and the required equipment is simple and controllable and it is easy to automate the management [13,16]. Most of the previously reported literature uses Fenton technology to treat water pollution [17]. However, the Fenton reaction needs to be carried out in an acidic environment at pH 3. At pH > 4, Fe^2+^ hydrolyzes to form iron hydroxide precipitates, and at pH < 2.5, the presence of H_2_O_2_ in the form of H_3_O_2_^+^ reduces the reactivity with Fe^2+^, which also leads to the occurrence of side reactions and reduces the efficiency of the reaction [17,18].

Two-dimensional (2D) nanomaterials, layered double hydroxides (LDHs) [19], have a variety of unique properties and have been used in diverse applications such as biosensors [20], supercapacitors [21], etc. Recently, they have attracted a lot of attention from researchers as electrocatalysts for oxygen evolution and reduction reactions, and have mostly been used as non-homogeneous catalysts for electro-Fenton-degradable wastewater treatment [22]. However, the intrinsic weak electrical conductivity and poor mechanical properties of LDHs impeded their further development [23,24]. Therefore, there is a need to composite LDHs with other materials, such as carbon. While it is common practice to load LDHs directly onto the surface of carbon materials, which significantly improves the conductivity of LDHs, the internal layered structure cannot effectively participate in the electrochemical reactions [24]. To address this, LDHs are exfoliated and self-assembled with exfoliated GO through electrostatic means, resulting in the construction of 2D/2D composites. These composites possess an interconnected network structure with a large specific surface area, which not only reduces the agglomeration of LDHs but also improves their electrical conductivity and enhances the utilization of LDHs. In this study, the electrocatalytic oxygen reduction (ORR) technology for H_2_O_2_ synthesis was coupled with the Fenton reaction, which overcame the problem of H_2_O_2_ and Fe^2+^ excretion in the traditional Fenton method. Herein, graphene was inserted into layered bimetallic hydroxide to form various LDH/rGO composites. Subsequently, the prepared LDH/rGO composites were loaded onto carbon felts, serving as electro-Fenton cathode materials. Using ceftazidime as the target pollutant, we aimed to investigate the effects of different metal species and morphologies, among other factors, on enhancing the performance of electrocatalytic oxygen reduction for the synthesis of H_2_O_2_.

## 2. Results and Discussion

### 2.1. Characterizations

#### 2.1.1. FT-IR and XRD Analysis

**Figure 1:**
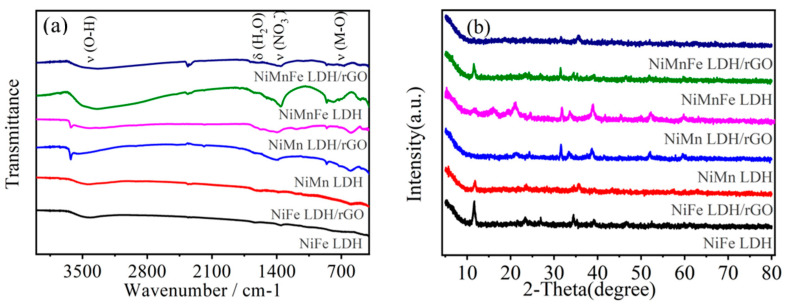
(**a**) FT-IR spectra and (**b**) XRD patterns of NiFe-LDH, NiFe-LDH/rGO, NiMn-LDH, NiMn-LDH/rGO, NiMnFe-LDH, and NiMnFe-LDH/rGO.

The FT-IR spectra of the prepared catalysts NiFe-LDH, NiFe-LDH/rGO, NiMn-LDH, NiMn-LDH/rGO, NiMnFe-LDH, and NiMnFe-LDH/rGO are shown in Figure 1a. NiFe-LDH, NiMn-LDH, and NiMnFe-LDH all show a peak at 3427 cm^−1^. The 3427 cm^−1^ broad absorption peaks are caused by the O-H tensile vibration, and the peak at 1630 cm^−1^ is the tensile bending vibration peak of O-H. The vibrational absorption peaks of the three LDHs/rGO at about 1703 cm^−1^, 1623 cm^−1^, 1224 cm^−1^, and 1056 cm^−1^ correspond to the C=O stretching vibration, the C=C stretching vibration, the C-OH stretching. The characteristic peaks at around 1357 cm^−1^ correspond to the vibrational absorption peaks of NO_3_^−^, while the characteristic peaks below 800 cm^−1^ are mainly the vibrational peaks of Ni-O, Fe-O, Mn-O, etc., which are clearly manifested as a generalized intensity of the characteristic peaks on the LDHs/rGO catalysts. The emergence of these new peaks proves the effective combination of LDHs and rGO. These characteristic peaks on LDHs/rGO catalysts obviously showed a general decrease in the intensity of characteristic peaks, which was due to the changes brought by the introduction of rGO.

Figure 1b illustrates the XRD spectra of several catalysts: NiFe-LDH, NiFe-LDH/rGO, NiMn-LDH, NiMn-LDH/rGO, NiMnFe-LDH, and NiMnFe-LDH/rGO. For NiFe-LDH, seven characteristic diffraction peaks are found at 2θ of 11.513°, 23.143°, 34.195°, 38.713°, 46.084°, 59.387°, and 60.720°, which correspond to the (003), (006), (012), (015), (018), (110), and (113) crystal planes of NiFe-LDH, respectively [25], indicating that the double hydroxide formed the desirable level structure. The entire baseline of the plots is relatively smooth, proving the good crystallinity of NiFe-LDH. For NiFe-LDH/rGO, since LDH and rGO are self-assembled in layers, it can be found that all seven characteristic peaks mentioned above are retained, but the diffraction peaks of rGO are not very obvious. A similar trend is observed in the spectra of NiMn-LDH and NiMn-LDH/rGO. For NiMnFe-LDH, it can be found that the characteristic peaks are exactly between those of NiFe-LDH and NiMn-LDH, indicating that the ternary LDH is well formed; in addition, the diffraction peaks of NiMnFe-LDH/rGO are obviously weaker than those of NiMnFe-LDH, which is attributed to the reduced crystallinity due to the presence of rGO. The GO bands were not obvious either, as shown in Figure S3 (Supplementary Materials). As we know, the XRD pattern of pristine GO presents a strong (001) peak at 2θ = 11.4; after the chemical reduction, the typical diffraction peak of rGO appears at 23.19 with a broad peak, and no other diffraction peaks are observed. In addition, the characteristic diffraction peak at 2θ of 11.513° and 23.143° was corresponding to the (003) and (006) crystal planes of LDHs. When LDH is assembled with GO, it may result in the shielding of graphene bands [26].

Raman spectroscopic analysis is employed to further confirm the composition of the samples. In Figure S4 (Supplementary Materials), there two Raman bands appear at 1590 cm^−1^ (G band) and 1328 cm^−1^ (D band) for the rGO sample, which are assigned to the sp^2^ carbon domains and the sp^3^ carbon atoms, respectively. The relative intensity ratio (I_D_/I_G_) of the D and G bands is usually employed to reflect the defect level in graphene [27]. It is calculated that the I_D_/I_G_ ratios were slightly higher in the reduced materials LDH/rGO, i.e., NiFe-LDH/rGO, NiMnFe-LDH/rGO, and NiMnFe-LDH/rGO than in NiFe-LDH/GO, NiMn-LDH/GO and NiMnFe-LDH/GO, indicating the increased crystal disorder during the chemical reduction of GO [28].

The proportions of all the key elements of LDHs/GO and LDHs/rGO from XPS analysis are listed in Table S3. Compared with LDHs/GO, it is obvious to observe that the content of element C in their corelated LDHs/rGO is sharply increased (from about 40 at% to 70 at%), whereas the O content was significantly decreased (from about 40 at% to 20 at%), showing that GO in the NiFe LDH/GO, NiMn LDH/GO, and NiMnFe LDH/GO is partially reduced to generate NiFe LDH/rGO, NiMn LDH/rGO, and NiMnFe LDH rGO.

#### 2.1.2. SEM Analysis

**Figure 2:**
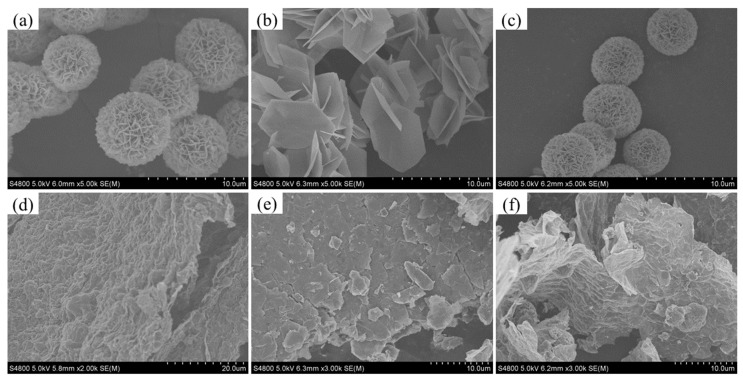
SEM images of different catalysts: (**a**) NiFe LDH, (**b**) NiMn LDH, (**c**) NiMnFe LDH, (**d**) NiFe LDH/rGO, (**e**) NiMn LDH/rGO, and (**f**) NiFeMn LDH/rGO.

The morphological features of the materials can be clearly observed by using scanning electron microscopy. It can be seen that pure GO has a layered structure with large surface wrinkles (Fiure S5 Supplementary Materials). Figure 2a–f showed that the three kinds of LDHs, NiFe LDH, NiMn LDH, and NiMnFe LDH, all exhibit a lamellar structure, which possesses the remarkable features of LDH. These LDHs are formed into three kinds of catalysts, namely, NiFe LDH/rGO, NiMn LDH/rGO, and NiFeMn LDH/rGO, after compounding with rGO, which shows that the catalysts display an obvious graphene irregular folding morphology, indicating that the LDH materials are well composited with rGO. Compared with LDHs, the composites possess a higher clarity, indicating that their electrical conductivity has been significantly enhanced. Overall, the LDH and LDH/rGO composites are well prepared and formed with ideal morphology characteristics.

Specifically, as shown in Figure 2a, NiFe LDH exhibits a porous spherical morphology constructed from loosely layered nanosheets. The diameter of these “balls” ranges from 5 to 8 μm, while the thickness of the nanosheets measures approximately 70 nm. After ultrasonic exfoliation and then self-assembly, it is found that the nanosheets cover the surface of rGO. The NiMn LDH is composed of hexagonal nanosheets assembled into small clusters in different ways; the size of the nanosheets is larger, about 2–3 μm, for NiMn LDH/rGO, and the nanosheets can also be found to cover the surface of rGO. The morphology of NiMnFe LDH is similar to that of NiFe LDH; it is also assembled into a spherical structure with a narrow slit, and the “ball” has a diameter of 5–8 μm. The morphology of NiMnFe LDH is comparable to that of NiFe LDH, as it assembles in a slit-like manner to create a spherical structure. However, the diameter of this “sphere” is slightly smaller, approximately 4–6 μm. Furthermore, the wrinkled shape of NiMnFe LDH/rGO can also be clearly observed.

#### 2.1.3. TEM Analysis

**Figure 3:**
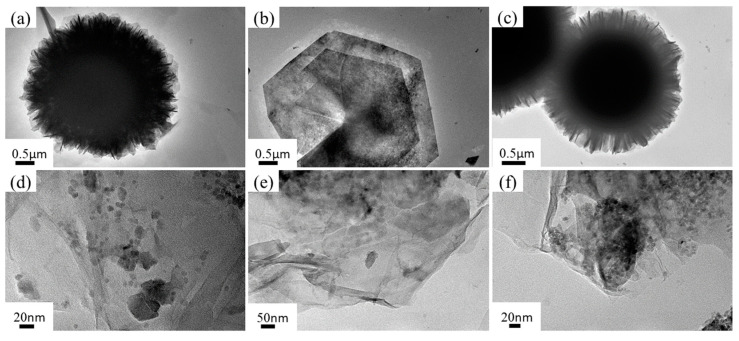
TEM morphology of different catalysts: (**a**) NiFe LDH, (**b**) NiMn LDH, (**c**) NiMnFe LDH, (**d**) NiFe LDH/rGO, (**e**) NiMn LDH/rGO, and (**f**) NiMnFe LDH/rGO.

The particle size and morphology of NiFe-LDH, NiFe-LDH/rGO, NiMn-LDH, NiMn-LDH/rGO, NiMnFe-LDH, and NiMnFe-LDH/rGO catalysts were investigated by using transmission electron microscope (TEM), which is shown in Figure 3. From Figure 3a, it can be seen that for NiFe-LDH catalysts, the lamellar structure is piled up in large quantities to form a shape similar to that of a hydrangea, with the diameter of the ball being about 4 μm; additionally, there is significant agglomeration inside the ball, and a large number of pore structures are formed at the edges due to the interspersing of the lamellae with each other. RGO can be found to have an obvious lamellar wrinkled structure and is disordered, as can be seen from Figure 3b. This is the typical morphology of the rGO structure. In addition, the NiFe-LDH lamellar structure is peeled off more thoroughly, and many lamellae are well dispersed on the supported lamellar wrinkled rGO sheets. NiMn-LDH has a smooth and uniform 2D nanosheet array structure with several lamellae stacked together, and the lamellae are large, with diameters of about 3–4 μm, and the average thickness is about 20 nm. From Figure 3c, it can be observed that the NiMn-LDH lamellae are better dispersed on the wrinkled rGO surface. The morphology of NiMnFe-LDH is very close to that of NiFe-LDH, which is also composed of a large number of lamellae stacked together to form a loose sphere; the surface lamellae of the ball are more sparse with a certain degree of porosity. The rGO of the NiMnFe-LDH/rGO structure has a remarkable lamellae wrinkled structure, which is not only a smooth and uniform two-dimensional nanosheet array structure, but also a smooth and uniform two-dimensional nanosheet array structure. The NiMnFe-LDH/rGO structure has a significant wrinkled lamellar structure, and the NiMnFe-LDH lamellae are dispersed on the surface of the wrinkled rGO, which effectively prevents the stacking and overlapping between the LDH lamellae.

The interpolating of graphene into the interlayers of LDHs is demonstrated by TEM in Figure S6 (Supplementary Materials). It can be explicitly seen from Figure S6a that the rGO nanosheets make contact with the NiFe-LDHs face to-face in NiFe LDH/rGO. To further obtain the internal structure characteristic of the NiFe LDH/rGO composite, Figure S6b gives a straightforward HRTEM observation; it can be clearly observed that there are two types of contrast fringes. Moreover, NiFe-LDHs and rGO are characterized by periodic assembly.

### 2.2. Electrocatalytic Oxygen Reduction Synthesis of H_2_O_2_ Performance

#### 2.2.1. LSV Analysis

**Figure 4:**
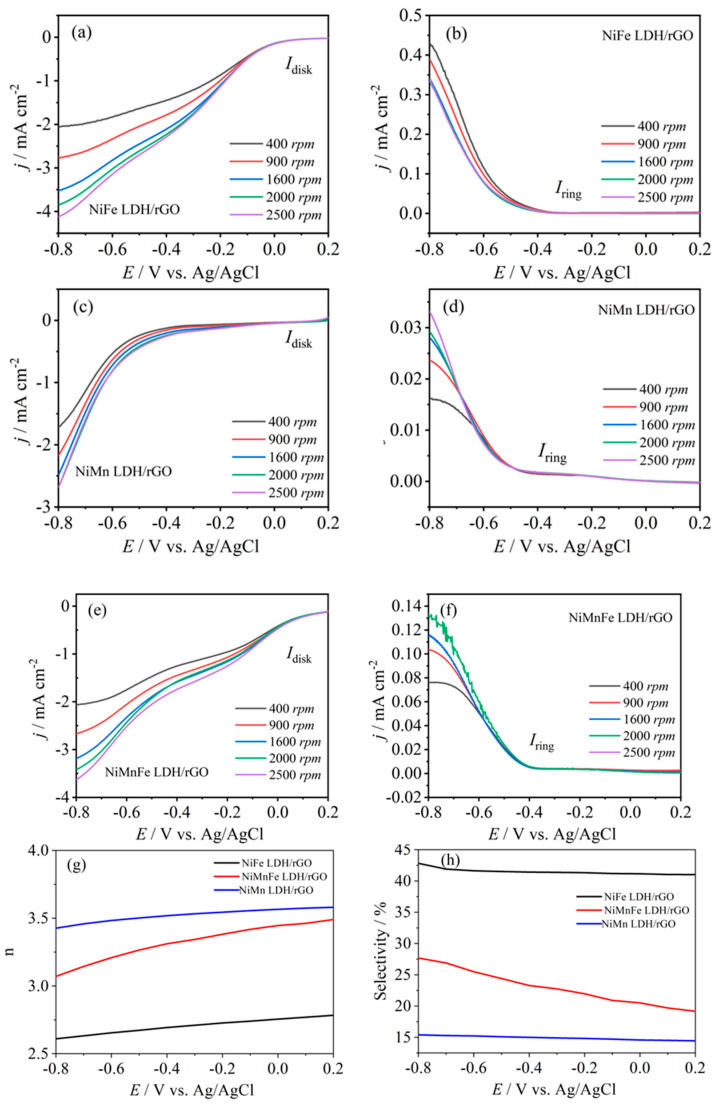
Disk (**a**,**c**,**e**) and ring (**b**,**d**,**f**) currents collected on bare RRDE at different rotation speeds, (**g**) number of transferred electrons, and (**h**) H_2_O_2_ selectivity of NiFe LDH/rGO, NiMn LDH/rGO, and NiMnFe LDH/rGO catalysts.

The electrocatalytic oxygen reduction (ORR) properties of the catalysts were evaluated using the rotating ring disk disc electrode (RRDE) technique to investigate and compare the activity and selectivity of three different catalysts, NiFe LDH/rGO, NiMn LDH/rGO, and NiMnFe LDH/rGO, in the electrocatalytic reduction of O_2_ to H_2_O_2_. Figure 4a–h shows the ORR disk currents, H_2_O_2_ oxidation ring currents, and H_2_O_2_ selectivity and electron transfer numbers in the potential range of −0.8–0.2 V vs. Ag/AgCl for the three catalysts at different rotational speeds, respectively. It was found that the ORR starting potential of the NiFe-LDH/rGO catalyst was about 0 V, which was close to the theoretical starting potential value of H_2_O_2_ produced by the electroreduction of O_2_ under acidic condition, and was more positive than the ORR starting potentials of the other catalysts (for the two catalysts NiMn-LDH/rGO and NiMnFe LDH/rGO, these were −0.55 and −0.05 V, respectively), indicating that the H_2_O_2_ synthesis by the electroreduction of O_2_ was achieved at a higher ORR current than the other catalysts were, suggesting that the synthesis of H_2_O_2_ by the electroreduction of O_2_ is more likely to occur over the NiFe-LDH/rGO catalyst.

In addition, the NiFe-LDH/rGO limiting disk current density was about 3.6 mA·cm^−2^ at 2500 rpm, exceeding that of the NiMn-LDH/rGO catalysts (~2.7 mA·cm^−2^) and slightly trailing the NiMnFe LDH/rGO catalysts (~4.1 mA·cm^−2^). The NiFe-LDH/rGO limiting ring current density was about 0.44 mA·cm^−2^, which was higher than that of NiMn-LDH/rGO and NiMnFe LDH/rGO catalysts (~0.035 mA·cm^−2^ and ~ 0.14 mA·cm^−2^, respectively). In the potential range of −0.8–0.2 V vs. Ag/AgCl, the H_2_O_2_ selectivity of the NiFe-LDH/rGO catalyst remains around 42%, while that of the other two catalysts is only 15–27%, indicating that the NiFe-LDH/rGO catalysts have a higher H_2_O_2_ selectivity. Meanwhile, by comparing the onset potential, current density, and H_2_O_2_ selectivity of the H_2_O_2_ catalysts, it was found that the H_2_O_2_ activity and selectivity of the electrically synthesized H_2_O_2_ from reduced O_2_ showed a volcano plot trend, and the NiFe-LDH/rGO catalysts had the optimal activity and selectivity of electrically synthesized H_2_O_2_. In addition, the electron transfer number of NiFe-LDH/rGO catalyst was about 2.7 (Figure 4g) and it can be considered that this catalyst mainly catalyzes the two-electron ORR reaction; meanwhile, the electron transfer number of NiMnFe LDH/rGO was about 3.1–3.3, and the electron transfer number of NiMn-LDH/rGO was about 3.5, which suggests that both catalysts catalyze the two-electron and four-electron ORR reactions, with the four-electron reaction being more dominant. These results indicate that the NiFe-LDH/rGO catalysts have better two-electron ORR activity and selectivity.

#### 2.2.2. Electrochemical Active Area Analysis

**Figure 5:**
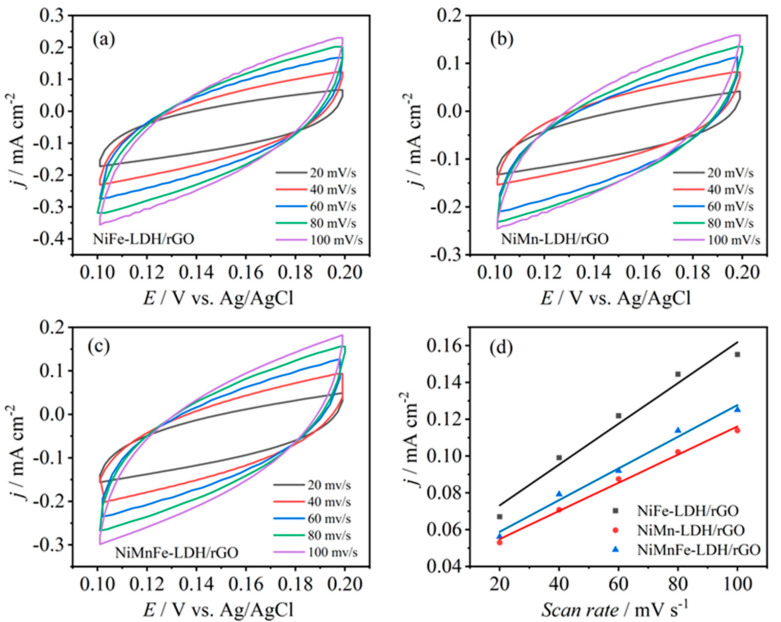
CV curves at different scan rate of (**a**) NiFe LDH/rGO, (**b**) NiMn LDH/rGO, and (**c**) NiMnFe LDH/rGO catalysts; (**d**) calculated double-layer capacitance. Note: CV curves are collected in the non-Faradaic potential region.

The performance of catalysts is usually related to their electrochemical active area (ECSA). We investigated the effect of ECSA on catalyst ORR activity by performing cyclic voltammetry tests in a 0.05 mol·L^−1^ Na_2_SO_4_ electrolyte with an initial pH of 3 at scanning speeds of 20, 40, 60, 80, and 100 mV·s ^−1^ to estimate the ECSA using the catalyst bilayer capacitance Cdl values. As shown in Figure 5, the Cdl of NiFe LDH/rGO, NiMn LDH/rGO, and NiMnFe LDH/rGO catalysts were 1 mF·cm^−2^, 0.7 mF·cm^−2^, and 0.8 mF·cm^−2^, respectively, and the ECSA of the catalysts was positively correlated with the Cdl value, so the ECSAs were ordered as NiFe LDH/rGO > NiMnFe LDH/rGO > NiMnFe LDH/rGO. rGO > NiMnFe LDH/rGO > NiMn LDH/rGO, which is almost the same as the ordering of catalyst ORR activity (onset potential and limiting current density), suggesting that a high ECSA is favorable for improving the ORR activity.

According to our previous BET results, the pore structure of the catalyst NiFe LDH/rGO is mainly dominated by mesopores, with the largest specific surface area, in comparison with NiMn LDH/rGO and NiMnFe LDH/rGO. The ECSA is related to the specific surface area, and especially to the mesopore-related specific surface area, so it is hypothesized that the ECSA may be mainly attributed to the specific surface area from the mesopores. Thus, the NiFe LDH/rGO catalyst with the largest specific surface area has the highest ECSA value.

In order to analyze NiFe LDH/rGO with the best two-electron ORR performance, the ORR performance of two- and four-electron pathways on various metal surfaces was calculated by Nørskov et al. using the DFT method [29]. Ni binds weakly to *OOH and needs to overcome an energy barrier of 0.18 eV for the formation of *OOH, which exhibits a weak two-electron ORR activity. Conversely, Fe or Mn binds strongly to *OOH, exhibiting strong four-electron ORR activity due to O–O rupture. Therefore, the binding energy of *OOH is optimized by modulating the electronic properties of Ni and Fe or Ni and Mn to favor the catalytic two-electron ORR. According to the effect of electronic structure, the morphology and size of the nanomaterials also affect the catalyst performance [30]. NiFe LDH/rGO has the largest specific surface area and ECSA value, which leads to the superior electrocatalytic activity.

### 2.3. Optimization of Electro-Fenton Process Conditions

#### 2.3.1. Effect of Initial Concentration

Generally speaking, the concentration of antibiotics in wastewater treatment plants shows periodic or seasonal fluctuations, and the concentration of antibiotics contained in wastewater from different sources varies, so it is of practical significance to study the effect of the initial concentration of antibiotics on the removal efficiency of the electro-Fenton process. High initial concentration, on the one hand, will lead to an increase in system resistance; in addition, mass transfer rate is reduced, mass transfer resistance increases, and current decreases. The rate of electron transfer is limited, on the other hand, under the same conditions; the ability to generate oxidatively active species per unit of time is limited, the target antibiotics will compete with each other when the concentration of the antibiotic is high, and at the same time, a large number of intermediary products generated during the reaction process will also participate in the competition, resulting in a decrease in the removal rate of the target antibiotics. The target antibiotics near the electrode are degraded, while the surrounding antibiotics are not replenished in time, so the solution will show a certain concentration gradient along the direction of the electrode, and the concentration polarization will lead to a decrease in the removal efficiency. In order to investigate the effect of initial concentration on the removal effect of ceftazidime, the pH value was fixed at pH = 3, the concentration of electrolyte Na_2_SO_4_ was 0.05 mol·L^−1^, the current density was 10 mA·cm^−2^, and the initial concentration of ceftazidime was taken as 2, 5, 10, and 20 mg·L^−1^.

**Figure 6:**
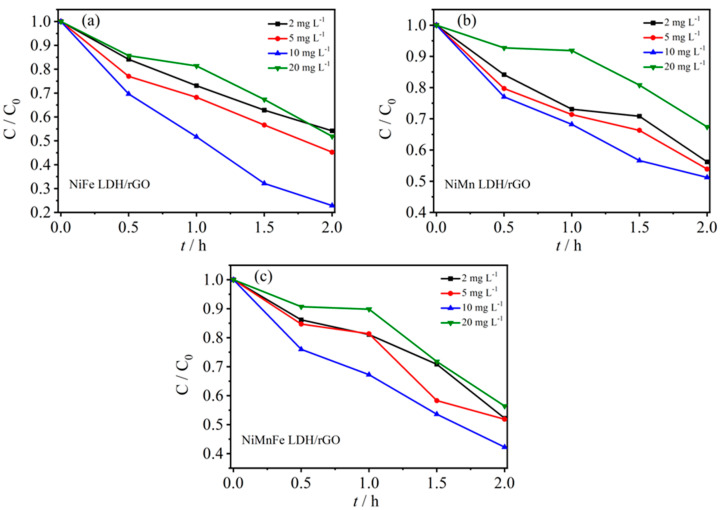
Effect of initial concentration on degradation of ceftazidime: (**a**) NiFe LDH/rGO, (**b**) NiMn LDH/rGO, and (**c**) NiMnFe LDH/rGO.

Figure 6a–c shows the effect of initial concentration on the efficiency of ceftazidime degradation by electrofenton at three LDH/rGO modified cathodes. For the NiFe LDH/rGO cathode material, the degradation rate of ceftazidime was 45% at an initial concentration of 2 mg·L^−1^; it increased to 55% when the initial concentration was increased to 5 mg·L^−1^, and reached a maximum of 78% when the initial concentration was increased to 10 mg·L^−1^, but decreased to 49% when the initial concentration was further increased to 20 mg·L^−1^. For the NiMn LDH/rGO and NiMnFe LDH/rGO cathodes, the degradation rate of ceftazidime increases and then decreases with the increase of the initial concentration, and the degradation rate reaches a maximum at the initial concentration of 10 mg·L^−1^, which is 45% and 58%, respectively. It can be assumed that when the initial concentration of ceftazidime is low, the oxidatively active species ·OH produced by the electrode per unit time has enough capacity to carry out electro-Fenton degradation of the target pollutants; however, higher initial concentration means that too much ceftazidime is competing with itself, and this, together with the accumulation of a large number of intermediate by-products, results in a decrease in the degradation rate.

#### 2.3.2. Effect of Solution pH

**Scheme 1:**
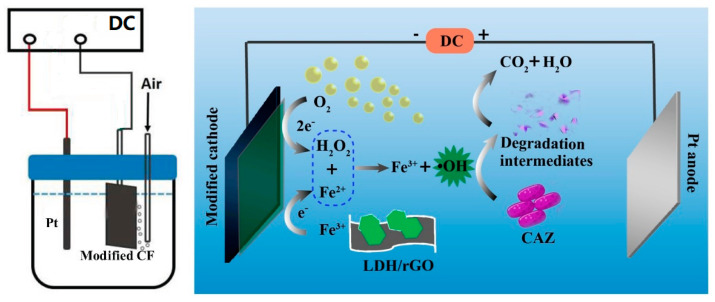
Electro-Fenton equipment (**left**); schematic illustration of heterogeneous electro-Fenton degradation of pollutants (**right**).

The electro-Fenton device and the schematic diagram of the principle are shown in Scheme 1. It is speculated that the mechanism of the bifunctional catalyst LDHs/rGO electro-Fenton reaction is as follows. Firstly, the oxygen molecules are adsorbed in the carbon sites of reduced graphene oxide and carbon felt, which are reduced to H_2_O_2_ by two-electron ORR; the generated H_2_O_2_ is then catalyzed by Fe(II) on the surface of the LDHs to generate ·OH, and the generated Fe(III) is rapidly reduced to Fe(II) due to the enhancement of the interfacial electron transfer between the graphene and LDHs, thus improving the efficiency of non-homogeneous phase electro-Fenton degradation. Due to the enhanced electron transfer at the interface between graphene and LDHs, the generated Fe(III) is rapidly reduced to Fe(II), which improves the efficiency of non-homogeneous electro-Fenton degradation of pollutants.

Solution pH is an important factor affecting the electro-Fenton degradation process, and the generation of oxidatively active species such as ·OH in the system, the occurrence of side reactions such as oxygen precipitation reactions, and the dissociation constants of the target pollutants are all affected by pH, which, in turn, affects the degradation rate of the pollutants. In addition, acidic or alkaline environments affect the number of charged particles in solution, the migration of pollutants in solution between electrodes, the mass transfer diffusion capacity, and the adsorption of pollutants by electrodes [31]. In order to investigate the effect of initial solution pH on the removal efficiency of ceftazidime, the concentration of electrolyte Na_2_SO_4_ was fixed at 0.05 mol/L, the initial concentration of ceftazidime was 10 mg·L^−1^, and the current intensity was 5 mA·cm^−2^, and the differences in the removal effect of cephalosporin were investigated from the conditions of acidic to alkaline media (pH = 3 to 9) (Na_2_SO_4_ at a concentration of 0.05 mol·L^−1^ was used as the electrolyte in a solution volume of 150 mL [solution pH was adjusted by pH meter with 0.5 mol·L^−1^ H_2_SO4 or NaOH]).

**Figure 7:**
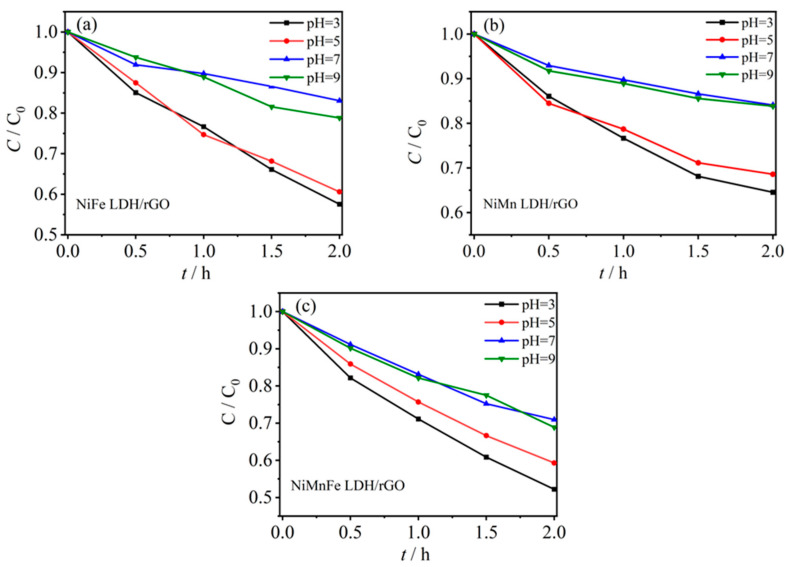
Effect of solution pH on degradation of ceftazidime: (**a**) NiFe LDH/rGO, (**b**) NiMn LDH/rGO, and (**c**) NiMnFe LDH/rGO.

Figure 7a–c shows the effect on the efficiency of ceftazidime degradation in the non-homogeneous electro-Fenton system at different pH conditions for three LDH/rGO modified electro-Fenton cathodes. For the NiFe LDH/rGO cathode material, the degradation of ceftazidime slightly decreased from 43% to 40% when the initial pH was increased from 3.0 to 5.0, and the removal of ceftazidime drastically decreased to 15–20% with the further increase of pH from 5.0 to 9.0. For NiMn LDH/rGO and NiMnFe LDH/rGO cathode materials, the overall trend shows that the degradation rate of ceftazidime decreases with the increase of pH, and the degradation rate slightly decreases when the pH increases from 3.0 to 5.0, which can be regarded as the cathode materials prepared in this paper increasing the pH range of the electro-Fenton degradation process. The degradation rate of the former is 36% and that of the latter is 48% at pH = 3. It can be found that the maximum degradation rate is found at pH = 3, which is consistent with the common belief that the Fenton reaction needs to be carried out in an acidic environment at pH = 3 [17]. When pH < 2.5, H_2_O_2_ exists in the form of H_3_O_2_^+^, which decreases the reactivity with Fe^2+^ and also leads to the occurrence of side reactions, reducing the efficiency of the reaction. An alkaline environment is not conducive to the formation of H_2_O_2_ and ·OH due to the low concentration of H^+^ ions, nor to the hydrolysis of Fe^2+^ to form iron hydroxide precipitates. In addition, the pH value of the solution also determines the presence form of antibiotics, such as the dissociation of antibiotics into cationic, anionic, or amphoteric forms. Due to the complex chemical structure of cephalosporin antibiotics such as β-lactam and dihydrothiazine rings, which have different functional groups and multiple ionizable parts, such as carboxyl groups, amino groups, and so on, the dissociation constant of ceftazidime pKa1 is 2.91 and that of ceftazidime pKa2 is 3.81 [32]. it can be found that the deprotonation of ceftazidime is greater under more acidic conditions, which lead to the faster oxidative degradation of ceftazidime.

#### 2.3.3. Effect of Current Density

Current density is also an important factor affecting the electro-Fenton degradation process. On the one hand, because the current density is closely related to the generation of oxidizing active species in the system, the current density is too low in the system of oxidizing active species yield and production will be on the low side, which affects the degradation efficiency of the pollutants; for the direct electro-oxidation, the current density is too low to limit the amount of electrons between the electrodes and the target pollutants and the electron transfer rate. On the other hand, a too-high current density is also accompanied by the occurrence of a large number of side reactions such as oxygen precipitation reaction and electrolyte decomposition, which lead to the reduction of current efficiency and a significant increase in energy consumption; at the same time, it is inevitable that the high current density will increase the loss of electrode materials and shorten the service life of the electrodes dramatically [33]. To investigate the effect of current density on ceftazidime degradation, the pH value was fixed at pH = 3, the electrolyte Na_2_SO_4_ concentration was 0.05 mol·L^−1^, the initial concentration of ceftazidime was 10 mg·L^−1^, and the current densities were 5, 10, 15, and 20 mA·cm^−2^, respectively.

**Figure 8:**
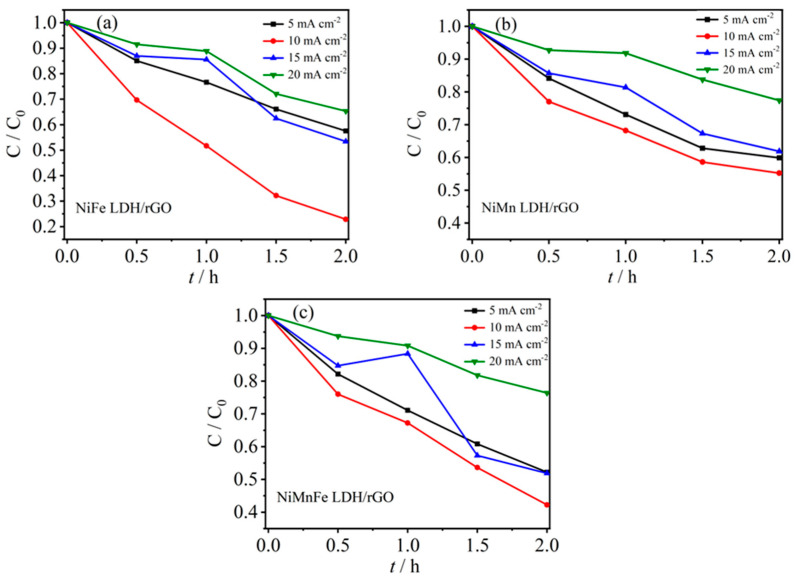
Effect of current density on degradation of ceftazidime: (**a**) NiFe LDH/rGO, (**b**) NiMn LDH/rGO, and (**c**) NiMnFe LDH/rGO.

Figure 8a–c shows the effect of current density on the efficiency of ceftazidime degradation by electrofenton at three LDH/rGO modified cathodes. For the NiFe LDH/rGO cathode material, the degradation of ceftazidime was 43% at a current density of 5 mA·cm^−2^, which significantly increased to 78% when the current density was increased to 10 mA·cm^−2^; however, it decreased to 45% and 35% when the current density continued to increase to 15 mA·cm^−2^ and 20 mA·cm^−2^. For the NiMn LDH/rGO, NiMn LDH/rGO, and NiMn LDH/rGO cathode materials, the degradation was 43%, which increased to 78% at a current density of 5 mA·cm^−2^ but decreased to 45% and 35%, respectively. For the NiMn LDH/rGO and NiMnFe LDH/rGO cathodes, the degradation rate of ceftazidime increases and then decreases with the increase of current density, and the degradation rate reaches the maximum at a current density of 10 mA·cm^−2^, which is 45% and 58%, respectively. This indicates that with the increase of current density, the transfer of electrons to H_2_O_2_ through the 2-electron ORR will be accelerated, as shown in Equations (1) and (2), so that the accumulation of H_2_O_2_ can be improved; thus, the content of ·OH in the active species can be increased, which, finally, causes a significant increase in the degradation rate. However, with the continued increase of current density, side reactions will also be generated, such as the production of four-electron ORR H_2_O and the decomposition of H_2_O_2_, as shown in Equations (3)–(5); the reactions inhibited ·OH production and ceftazidime degradation.

O_2_ + e^−^ → ·O_2_^−^/·HO_2_^−^ (1)

·O_2_^−^ + 2 H+ + e^−^ → 2 H_2_O_2_ (2)

O_2_ + 4H+ + 4e^−^ → 2 H_2_O (3)

H_2_O_2_ → HO_2_· + H^+^ + e^−^ (4)

H_2_O_2_ + ·OH → H_2_O + ·O_2_H (5)

In addition, the increase in current density will also lead to an increase in side reactions such as oxygen precipitation, while the gas generated by the side reactions may adhere to the surface of the electrode to form a layer of gas, hindering the contact between the target pollutants and the electrode, thus affecting the degradation rate. Increasing the current density may also increase the temperature of the solution in the reactor, resulting in energy loss and higher energy consumption as well as accelerating the loss of the electrode’s active substances, thus shortening the service life of the electrode. The service life of the electrodes was also shortened by accelerating the depletion of the electrode active substances. In summary, 10 mA·cm^−2^ was determined as the optimal reaction current density.

The exploration of the electro-Fenton process conditions resulted in the optimal degradation conditions of pH = 3, electrolyte Na_2_SO_4_ concentration of 0.05 mol·L^−1^, current density of 10 mA·cm^−2^, and initial concentration of ceftazidime of 10 mg·L^−1^, respectively.

#### 2.3.4. Stability Analysis of Electro-Fenton Cathode Materials

**Figure 9:**
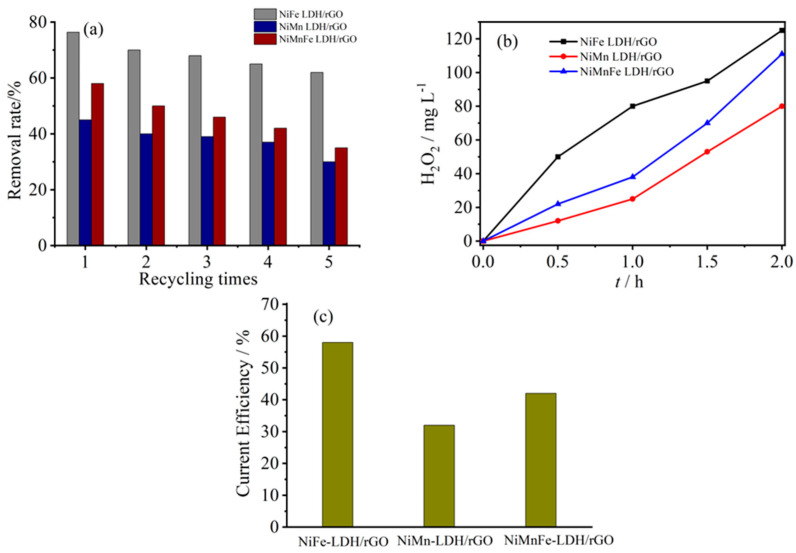
(**a**) Reproducible experiments of NiFe LDH/rGO, NiMn LDH/rGO, and NiMnFe LDH/rGO, three cathode materials, on degradation of ceftazidime, (**b**) current efficiency, and (**c**) cumulative concentration of hydrogen peroxide.

Stability is a very important index to examine the practical application of electrode materials. We investigated the reusability of the electrode materials by subjecting the three LDH/rGO-modified cathode materials prepared and obtained to ceftazidime to five successive repetitions of the electro-Fenton degradation process and examined the trend of the removal efficiency of the target pollutants. The experiments were carried out under the optimal degradation conditions, i.e., pH 3, electrolyte Na_2_SO_4_ concentration of 0.05 mol·L^−1^, current density of 10 mA·cm^−2^, and an initial concentration of ceftazidime of 10 mg·L^−1^. The removal rate of ceftazidime was determined by taking a sample of water after 2 h of degradation in order to examine whether the prepared cathode material could maintain a high removal rate of ceftazidime under the experimental conditions in a long-term operation. The removal rate of ceftazidime was determined by taking water samples after 2 h of degradation. As shown in Figure 9a, for the NiFe LDH/rGO cathode material, the degradation efficiency of ceftazidime gradually decreased from the initial 78% to 70% after five successive repetitions of electro-Fenton-degradation process, which may be attributed to the adsorption of ceftazidime and its intermediates on the surface of the electrodes, which led to the inactivation of some active sites. However, the degradation efficiency was still maintained at about 90%, indicating that the NiFe LDH/rGO electrode has good stability after many uses; for the NiMn LDH/rGO electrode, the degradation rate of ceftazidime decreased significantly after repeating the electro-Fenton experiment five times and only about 70% was retained, whereas the degradation rate of the NiMnFe LDH/rGO electrode was maintained at 80%. Comparing these three electrode materials, NiFe LDH/rGO has the best stability. The stability of the catalyst may be attributed to the strong interaction between graphene and NiFe LDH and the effective prevention of iron dissolution by graphene as a protective layer of NiFe LDH.

Figure 9b shows the accumulation of H_2_O_2_ of the three cathode materials under the optimal conditions, from which it can be seen that after 2 h, the accumulation of H_2_O_2_ of the NiFe LDH/rGO cathode material reaches 30 mg·L^−1^; meanwhile, the accumulation of H_2_O_2_ of the NiMn LDH/rGO cathode material reaches 22 mg·L^−1^, and that of the NiMn LDH/rGO cathode material is 17 mg·L^−1^. This indicates that the H_2_O_2_ accumulation of the NiFe LDH/rGO cathode material is the largest under the same conditions, which also provides the basis for the subsequent activation of H_2_O_2_ into ·OH to achieve more degradation of ceftazidime.

The current efficiencies of the three cathode materials under optimal conditions are shown in Figure 9c, from which it can be observed that NiFe LDH/rGO is about 58%, while NiMn LDH/rGO and NiMnFe LDH/rGO are 35% and 40%, respectively. The high current efficiency indicates that this electrosynthesis H_2_O_2_ process represents a highly efficient electrical-to-chemical energy conversion, and it also implies that more ceftazidime is degraded by the NiFe LDH/rGO cathode material under the same conditions in the same amount of time. The performance of the current work is compared to that of other previously published work [34,35,36,37] in Table S2; we find that LDH/rGO, especially NiFe LDH/rGO, has better electro-Fenton degradation properties.

## 3. Experimental Section

### 3.1. Experimental Materials

All the reagents in this study were analytic grade and commercially available. Graphite powder was purchased from Sigma-Aldrich (Shanghai, China). H_2_O_2_ was acquired Sinopharm Chemical Reagent Co. (Shanghai, China) ceftazidime (pharmacology) was purchased from Shanghai yuanye Bio-Technology Co., Ltd. (Shanghai, China) Ni(NO_3_)_2_∙6H_2_O, Mn(NO_3_)_2_∙4H_2_O, Fe(NO_3_)_3_∙9H_2_O, urea, NH_4_F, formamide, and other reagents were bought from Shanghai Aladdin Biotech Co., Ltd. (Shanghai, China) and used directly.

### 3.2. Preparation of LDH

The LDH was prepared by hydrothermal method. For nickel–manganese–iron LDH, the preparation method was as follows: firstly, 0.1939 g (0.667 mmol) of nickel nitrate hexahydrate, 0.1674 g (0.667 mmol) of manganese nitrate tetrahydrate, 0.2695 g (0.667 mmol) of iron nitrate hydrate nonahydrate (the total amount of control metal was 2 mmol), 0.2963 g (8 mmol) of ammonium fluoride, and 0.6006 g (10 mmol) of urea were weighed in a beaker and dissolved in 60 mL of deaerated distilled water; the mixture was transferred to a 100 mL reactor, and the reaction was carried out at 120 °C for 8 h. After the reactor was cooled down to room temperature, the product was washed by centrifugation with degassed distilled water and ethanol three times, and centrifugation was performed at 4000 r/min for 5 min/time. The washed solid product was filtered (0.22 μm membrane) and transferred into a beaker, part of which was directly used for subsequent stripping, and the other part was dried under vacuum at 60 °C for 12 h. The obtained product was NiMnFe-LDH.

For NiFe-LDH, only the amount of metal salt in the preparation method needs to be adjusted to 0.2908 g (1 mmol) of nickel nitrate hexahydrate and 0.4040 g (1 mmol) of iron nitrate nonahydrate, and the rest of the steps are the same as above.

For NiMn-LDH, only the amount of metal salt in the preparation method needs to be adjusted to 0.2908 g (1 mmol) of nickel nitrate hexahydrate and 0.2510 g (1 mmol) of manganese nitrate tetrahydrate, and the rest of the steps are as above.

### 3.3. Stripping of LDH

The paste obtained after hydrothermal pumping and filtration was weighed 0.1 g, dispersed in 100 mL of degassed formamide, and ultrasonicated using an ultrasonic crusher (~80% power) to strip the LDHs, and after 120 min of intermittent ultrasonication the stripped LDH suspensions were obtained, which were recorded as LDHs, NiMnFe-LDHs, NiFe-LDHs, and NiMn-LDHs.

### 3.4. Preparation of LDHs/rGO Composites

Graphene oxide was prepared using the modified Hummers method [25] and described in the Supplementary Materials. Then, 30 mg of monolayer graphene oxide was dispersed into 100 mL of formamide and ultrasonicated for 30 min until the formation of graphene oxide homogeneous colloid. The GO homogeneous colloid and the LDHs were placed in two constant-pressure dropping funnels and slowly dripped into a three-necked flask while stirring with nitrogen; the same dripping speed was used for controlling both sides of the dripping, during which the positively charged LDHs’ laminae were electrostatically self-assembled with negatively charged GO. After sufficient stirring, the mixture was left for a period of time to make it settle. The mixed solution was stratified, and the precipitate was washed by centrifugation with distilled water and ethanol, three times each, and the centrifugation condition was 10,000 r/min for 10 min/time. The resulting solid product was transferred to a petri dish for freezing for at least 12 h, and then transferred to a freeze dryer for freeze drying.

**Scheme 2:**
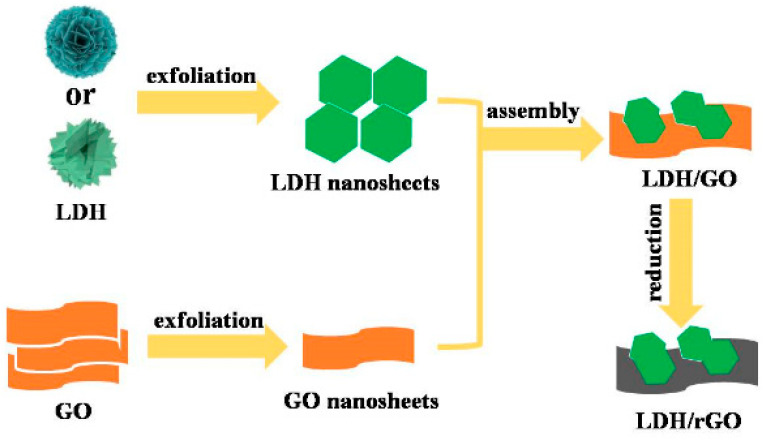
Schematic illustration of catalyst preparation process.

The solid product (LDHs/GO) obtained after freeze drying was taken as 50 mg ground and evenly dispersed in the bottom of a small glass vial, and the small glass vial was placed in a 100 mL reaction kettle with open mouth. Next, 500 μL of hydrazine hydrate was added to the kettle and the reaction was carried out for 12 h at 90 °C for the reduction. The samples were taken out of the kettle after it had cooled down to room temperature and the kettle was then dried in a vacuum at 60 °C for 12 h in order to remove the residual reductant. The LDHs/rGO were obtained as NiMnFe-LDHs/rGO, NiFe-LDHs/rGO, and NiMn-LDHs/rGO, respectively. The schematic diagram of the preparation is shown in Scheme 2.

## 4. Conclusions

LDHs were coupled with carbon materials to construct novel LDHs-based composites. Three composites, NiFe LDHs/rGO, NiMn LDHs/rGO, and NiMnFe LDHs/rGO, were successfully constructed by interpolating graphene into the interlayers of LDHs by an electrostatic self-assembly method. The prepared LDHs/rGO were loaded on carbon mats to construct an electro-Fenton cathode material, and the in situ electrocatalytic reduction of O_2_ to ·OH was realized as a non-homogeneous electro-Fenton oxidative degradation of organic pollutants. Using ceftazidime as a simulated pollutant, the electro-Fenton degradation of ceftazidime by three kinds of LDHs/rGO composites was investigated, and the effects of the electro-Fenton process conditions, such as the pH value of the solution, the concentration of the electrolyte, and the initial concentration of the antibiotic, on the pollutant removal rate were examined. The optimal degradation conditions were obtained as follows: pH = 3, electrolyte Na_2_SO_4_ concentration of 0.05 mol·L^−1^, current density of 10 mA·cm^−2^, respectively, with an initial concentration of ceftazidime of 10 mg·L^−1^. The reutilization rate of the material and the cumulative yield of hydrogen peroxide were examined under the optimal process conditions, and it was found that NiFe LDH/rGO had superior electro-Fenton-degradation. The effect of NiFe LDH/rGO was found to be excellent in the electro-Fenton degradation of pollutants. The use of non-homogeneous electro-Fenton degradation of wastewater, with its low cost and high catalytic efficiency, is convenient for practical application, which provides a new idea for solving difficult problems in the field of wastewater treatment.

## 5. Outlook

In the future, we want to investigate the effects of different compositional ratios of metal ions, as well as synthesis temperature and time, on the composition and structure (crystal shape, morphology, and size) of hydrotalcite. Then, we want to examine the effects of the exfoliation condition of electrostatic self-assembly and the intercalation content of graphene oxide on the modulation of morphology of hydrotalcite composites. Moreover, the mechanism of electro-Fenton degradation will be explored at the molecular level, which will provide scientific basis and theoretical guidance for the further establishment and development of the technology for the non-homogeneous electro-Fenton oxidative degradation of antibiotic wastewater. In the next stage, the examined aspects of different pollutants could be expanded, and the self-supporting cathode materials considered.

## Data Availability

Data are contained within the article.

## Supplementary Materials

The following supporting information can be downloaded at: https://www.mdpi.com/article/10.3390/molecules29133157/s1, Table S1. BET surface area and pore volume with different catalysts. Table S2. A comparison of the performance of the current work and previously published work. Table S3. Elemental proportions of NiFe LDH/GO, NiFe LDH/rGO, NiMn LDH/GO, NiMn LDH/rGO, NiMnFe LDH/GO, and NiMnFe LDH/rGO from XPS. Figure S1. The structure of ceftazidime. Figure S2. FT-IR spectra of NiMnFe LDH, NiMnFe LDH/GO, and NiMnFe LDH/Rgo. Figure S3. XRD patterns of (a) NiFe LDH/GO, NiMn LDH/GO, and NiMnFe LDH/GO, and (b) NiFe LDH, NiFe LDH/GO, and NiFe LDH/rGO. Figure S4. Raman spectra of NiFe LDH/GO, NiFe LDH/rGO, NiMn LDH/GO, NiMn LDH/rGO, NiMnFe LDH/GO, and NiMnFe LDH/rGO. Figure S5. SEM images of GO. Figure S6. TEM image (a) and HRTEM image (b) of NiFe LDH/rGO. Figure S7. EDS spectra of different catalysts. Figure S8. BET curves of NiFe LDH, NiMn LDH, NiMnFe LDH, NiFe LDH/rGO, NiMn LDH/rGO, and NiMnFe LDH/rGO. Figure S9. Wide XPS spectra of (a) NiFe LDH, NiMn LDH, and NiMnFe LDH catalysts, (b) NiFe LDH/GO, NiMn LDH/GO, and NiMnFe LDH/GO catalysts, (c) NiFe LDH/rGO, NiMn LDH/rGO, and NiMnFe LDH/rGO catalysts, and high-resolution scanning XPS spectra for (d) Ni 2p, (e) Fe 2p of NiFe LDH/rGO, and (f) Mn 2p of NiMnFe LDH/rGO. Figure S10. EIS plots of the electrocatalysts of NiFe LDH/rGO, NiMn LDH/rGO, and NiMnFe LDH/rGO.
