# Supplementary material for: Application of Bimetallic Hydroxide/Graphene Composites in Wastewater Treatment
Source: Molecules. 2024 Jul 2;29(13):3157. doi: 10.3390/molecules29133157 (PMC11243626; doi:10.3390/molecules29133157)
Supplement: Supplementary file 1 [file molecules-29-03157-s001.zip › molecules-3007625-supplementary.pdf]

Application of Bimetallic Hydroxide/Graphene Composites in Wastewater Treatment

Dan Chen [1,2,†], Jiao Wang [1,†], Nana Li [1], Xiaoqin Luo [1], Hua Yu [1,2], Haichang Fu [1,2], Zhangxin Chen [1,2,*], Binbin Yu [1,2,*], Yanxian Jin [1,*] and Dmitry S. Kopchuk [3]

[1] School of Pharmaceutical and Chemical Engineering, Taizhou University, Taizhou 318000, China; chendan824@126.com (D.C.); chunmianhua0525@163.com (J.W.); linana1640@163.com (N.L.); lxq18886201542@163.com (X.L.); yh624304@163.com (H.Y.); hcfu@tzc.edu.cn (H.F.)
[2] Taizhou Biomedical and Chemistry Industry Institute, Taizhou 318000, China
[3] Chemical Engineering Institute, Ural Federal University, 19 Mira Str., Ekaterinburg 620002, Russia; dkopchuk@mail.ru
* Correspondence: czx1992@tzc.edu.cn (Z.C.); yubinbin2004@126.com (B.Y.); snowflakej@163.com (Y.J.)
† These authors contributed equally to this work.

Reagents and instruments

Graphite powder was purchased from Sigma-Aldrich (Shanghai, China). H_2O_2 was acquired Sinopharm Chemical Reagent Co. ceftazidime (pharmacology) was purchased from Shanghai yuanye Bio-Technology Co., Ltd. $Ni(NO_3)_2{\cdot}6H_2O$, $Mn(NO_3)_2{\cdot}4H_2O$, $Fe(NO_3)_3{\cdot}9H_2O$, urea, NH_4F, formamide and other reagents were bought from Shanghai Aladdin Biotech Co., Ltd (Shanghai, China) and used directly.

The high performance liquid chromatography (HPLC) characterizations were performed on an Waters2695 spectrophotometer (Waters, USA). Ultraviolet–visible (UV-vis) were recorded using a DR5000 spectrometer (Hach, USA). The morphologies were acquired from a S-4800 emission scanning electron microscopy (Hitachi, Japan) and a F20 transmission electron microscopy (Hitachi, Japan). X-ray diffraction (XRD) characterization was conducted with a D8 Advance detector (Bruker, Germany). Fourier transform infrared spectra (FT-IR) was performed with a VERTEX 70 FTIR system (Thermo Fisher Scientific, Germany). Electrochemical impedance spectroscopy (EIS), cyclic voltammetry (CV), and linear scanning voltammetry (LSV) were obtained on an electrochemical analyzer (Chenhua, China). Brunauer-Emmett-Teller (BET) was obtained on an ASAP 2020 HD88 system (Micrometitics, USA). X-ray photoelectron spectrometer (XPS) characterizations were implemented on a Escalab 250Xi system (Thermo Fisher Scientific, USA). All electrochemical characterizations were implemented using a convenient three-electrode system: a rotating ring disc electrode (disk diameter 4.57 mm, ring outer diameter 5.38 mm, ring inner diameter 4.93 mm) as the working electrode, an Ag/AgCl as the reference electrode, and a platinum wire as the counter electrode.

2.1 Preparation of monolayer GO

1 g of graphite powder and 23 mL of concentrated sulfuric acid were stirred for half an hour in an ice bath (4-5°C). 3 g of potassium permanganate was added gradually under stirring for about 1h. Under this condition, the reaction was kept for 2 h. Then the temperature was warmed up to 50°C, 50 mL of deionized water was added slowly. Next, the mixture was raised to 90℃, and the reaction was continued for 1.5 h. After that, 20 mL of deionized water and 25.3 mL of H_2O_2 were added slowly until the reaction stopped. The solution centrifuged and washed for several time until the pH value of the supernatant was neutral. The filtration was dried in vacuum at 60 °C, the GO was obtained.

Then 0.1 g of dried GO were suspended in 100 mL dimethylformamide. After ultrasonicating for 3 h, the colloidal GO was prepared. Followed by centrifuging, the exfoliated colloidal GO nanosheets were obtained in the supernatant. The solid monolayer GO was obtained after freeze-drying.

2.2 Preparation of working electrode

The rotating ring disk electrode was sequentially polished to a mirror-like surface with Al_2O_3 polishing powder with a median particle size of 1.5 μm and 50 nm, washed with water and dried before use. 5 mg of catalyst was weighed in a centrifuge tube with an analytical balance, 50 μL of 5 wt % Nafion solution and 0.5 mL of anhydrous ethanol were added,

and the tube was sealed at the mouth, sonicated for 30 min until ink-like, and 20 μL of the suspension was applied to the surface of the rotating ring-disk electrode by pipetting with a pipette gun, and then dried to obtain the working electrode, which was loaded with 0.11 mg·cm^{-2} of catalyst.

2.3 Oxygen reduction performance testing of catalysts

When O_2 is reduced on the disk electrode, the product generated on the electrode rotating disk electrode is thrown out, and at the same time, a fixed potential is added to the ring electrode to detect the reaction product, i.e., to add a positive potential that can oxidize H_2O_2 but not water molecules, so as to study the selectivity of the catalyst for the production of H_2O_2 by the oxygen reduction reaction.

(1) LSV test

This was carried out in the potential interval of 0.2-0.8 V vs Ag/AgCl, with a scanning speed of 5 mV/s, rotating ring-disk electrode speeds of 400, 900, 1600, 2000, and 2500 rpm, and the ring electrode potential was controlled at 0.55 vs Ag/AgCl to obtain the polarization curves.

(2) Electrochemical active area (ECSA) test

Cyclic voltammetry (CV) tests were performed at 10, 20, 40, 60, and 80 mV·s^{-1} scanning rates in the potential interval of 0.2-0.8 V vs Ag/AgCl (no potential applied to the ring electrode), and the catalyst's bilayer capacitance, Cdl, was obtained by calculating the slope of the curve that fits the linearity of the current density versus the scanning rate ($\Delta J/2 = f(r)$), and the ECSA value is directly proportional to the Cdl value due to the fact that ECSA is proportional to the Cdl value. value is proportional to the value of Cdl, the value of ECSA was obtained.

(3) Electrochemical Impedance Spectroscopy (EIS) Analysis

The kinetic behavior of the catalyst was studied at open circuit voltage (no potential applied to the ring electrode) with an amplitude of 5 mV and a frequency of 0.1-10^5 Hz.

2.4 Electro-Fenton degradation experiment

2.4.1 Preparation of catalyst-modified carbon felt cathodes

Firstly, the carbon felt should be pre-treated and sonicated using the sequence of concentrated nitric acid, ultrapure water, acetone, ethanol, and ultrapure water to improve its wettability and remove surface impurities. After that, 5.0 mg of catalyst sample was taken, added with 0.5 mL of anhydrous ethanol and 50 μL of Nafion solution with 5% mass content, and ultrasonicated for 30 min to make it uniformly dispersed to obtain the catalyst suspension solution, and then pipetted 45 μL of the catalyst onto the carbon felt to be used as a cathode, and the loading of catalyst on the felt reached 0.4 mg·cm^{-2}.

2.4.2 Degradation device

The electro-Fenton degradation setup for this experiment consisted of a 200 mL single-port electrolytic cell, a magnetic stirrer, and a DC regulated power supply, with Na_2SO_4 at a concentration of 0.05 mol·L^{-1} as the electrolyte and a solution volume of 150 mL (initial pH of 3, solution pH was adjusted by 0.5 mol·L^{-1} H_2SO_4 and NaOH), platinum sheet (2 cm × 2 cm) and catalyst-modified Platinum sheet (2 cm × 2 cm) and catalyst-modified carbon felt (1 cm × 1 cm × 3 mm) electrodes were used as the anode and cathode, respectively, and the aeration head was placed near the cathode to provide oxygen, the flow rate of oxygen was controlled to be 0.6 L·min^{-1} , and the electrode spacing was 2 cm.

2.4.3 Steps of degradation experiments

Cephalosporin antibiotics (belonging to the beta-lactam group of antibiotics) occupy a large share of the antibiotics market and are the most commonly used antibiotics in the healthcare industry. Among them, ceftazidime is a third-generation cephalosporin antibiotic, commonly used in the treatment of respiratory, intestinal, and abdominal infections, with a broad spectrum and a long half-life, it is a cephalosporin that is used more frequently. With strong bactericidal effect, it is difficult to be decomposed by microorganisms directly and easy to accumulate in organisms, the structural formula is shown in Figure S1. Therefore, cephalosporin antibiotics are selected as target pollutants in this paper.

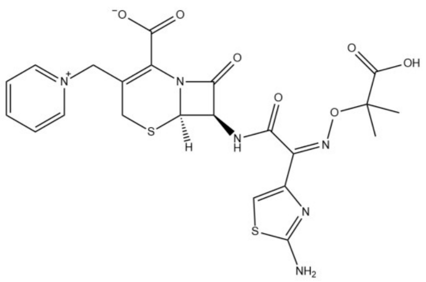

Figure S1. The structure of ceftazidime.

Firstly, configure ceftazidime reserve solution, weigh 100 mg ceftazidime directly dissolved in deionized water to make 100 mg·L^{-1} reserve solution, stored in the refrigerator at 4°C, due to the easier hydrolysis of β-lactam antibiotics, ceftazidime reserve solution was updated weekly.

Before each degradation experiment, the stock solution was diluted to the desired concentration (1-50 mg·L^{-1}), and samples were taken with a 1 mL disposable syringe every 30 minutes during the degradation process, and the concentration of the target pollutants was determined by a high performance liquid chromatography (HPLC) analyzer through a 0.22 μm glass-fiber filter tip in the mobile phase and wavelength range.

2.5 Analytical test methods

2.5.1 Methods for ceftazidime

In this paper, the concentration of ceftazidime during the reaction was determined by HPLC. The column was an Agilent TC-C18 column (250 mm×4.6 mm, 5 μm) with acetonitrile and KH_2PO_4 (6.8 g·L^{-1}), pH = 3.4 adjusted by H_3PO_4) at a flow rate of l mL·min-1 in a volume of 20 μL at a detection wavelength of 254 nm and a flow rate of 1 mL·min^{-1} at room temperature. The detection wavelength was 254 nm and the column temperature was room temperature. The column was washed with methanol and water in the ratios of 1:0 and 0.125:0.875, respectively, for 1 h before and after each measurement, and all mobile phases were filtered by 0.45 μm membranes.

Prior to the start of the degradation experiments, a series of standard solutions of ceftazidime with known concentration gradients were configured for the determination of the calibration line and the determination of the peak time, in which the peak time of ceftazidime was approximately 6.8 min. At the start of the degradation experiments, samples were taken at regular intervals with a 1 mL disposable syringe and measured.

2.5.2 Calculation of ceftazidime degradation rate and current efficiency

In this paper, the degradation rate of ceftazidime was used as an important index to measure the electrocatalytic performance of the electrode, and the degradation rate of ceftazidime was based on Equation (S1).

$$\eta\ (\%) = C / C_0 \quad \text{(S1)}$$

Where η is the degradation rate of ceftazidime, C0 and C are the initial concentration of the target pollutant and the concentration at time t, respectively, in mg·L^{-1}.

Current efficiency can visualize the efficiency of electrode utilization of current in the reaction system, which is another index to judge the performance of electrode. The current efficiency is calculated according to Equation (S2).

$$CE\ (\%) = FN/It \quad \text{(S2)}$$

where CE is the current efficiency of ceftazidime, F is Faraday's constant, N denotes the number of moles of ceftazidime (mol) participating in the reaction, I is the applied current (A), t denotes the electrolysis time (s).

2.5.3 Selectivity, electron transfer number and concentration determination of H_2O_2

The H_2O_2 selectivity and electron transfer number were calculated from the disk current measured at the rotating ring disk disc electrode as well as the ring current using the Equation (S3).

$$H_2O_2(\%) = 200 \times \frac{I_R/N}{I_D + I_R/N}$$

$$N = 4 \times \frac{4 \times I_D}{I_D + I_R/N} \quad \text{(S3)}$$

where I_D and I_R represent the disk and ring current densities (mA·cm^{-2}), respectively, and N is the H_2O_2 collection coefficient of the Pt ring, with a corrected value of 0.22. H_2O_2 selectivity is defined as the number of O_2 molecules reduced

to H_2O_2 as a percentage of the total number of O_2 molecules subjected to the reduction reaction during the electrocatalytic oxygen reduction process. n is the number of electrons transferred.

The quantitative analysis of H_2O_2 was determined by the potassium titanium oxalate method by UV-visible spectrophotometer. Firstly, the titanium reagent was prepared by adding 30.0 mL of deionized water to 27.2 mL of concentrated sulfuric acid, cooled down, and 3.54 g of $K_2TiO(C_2O_4)_2$ was added into the sulfuric acid solution, and then the volume was fixed to 100 mL. The method of testing was as follows: different concentration gradient of H_2O_2 standard solution (1-6 mmol·L^{-1}) was prepared separately, and 2.50 mL of the standard solution was added into a 10 mL colorimeter tube and 2.00 mL of titanium reagent was added into it. 2.50 mL of the standard solution was put into a 10 mL colorimeter tube, 2.00 mL of titanium reagent was added into the tube, the color was developed for 10 min, and then the solution was filtered through a 0.45 μm filter membrane and the absorbance was measured at 400 nm, and the absorbance-concentration standard curve was plotted.

Table S1. BET surface area and pore volumn with different catalysts.

Catalysts	BET surface area [a] (m^2g^{-1})	Pore volumn (cm^3g^{-1})
NiMnFe-LDH	38.7914	0.15
NiMnFe-LDH/rGO	26.3482	0.09
NiFe LDH	49.3165	0.19
NiFe LDH/rGO	35.1679	0.13
NiMn LDH	1.8948	0.005
NiMn LDH/rGO	0.9122	0.001

Table S2. A comparison of the performance of the current work and previously published work.

Adsorbate	Efficiency	pH	Reference
NiO-$NiFe_2O_4$-rGO	20.16 mg·L^{-1}	7	1
LDH/rGO/PANI/Au	0.5 μg/mL	7	2
S@NiFeLDH/rGO	1014 mAh g^{-1}	-	3
LDH/rGO	20%	-	4
LDH/rGO	58%	3	This work

Table S3. Elemental proportions of NiFe LDH/GO, NiFe LDH/rGO, NiMn LDH/GO, NiMn LDH/rGO, NiMnFe LDH/GO, and NiMnFe LDH rGO from XPS.

Sample	Elemental proportions / at %				
	C	O	Ni	Fe	Mn
NiFe LDH/GO	49.93	39.76	7.25	3.06	
NiMn LDH/GO	40.31	42.3	16.1		1.29
NiMnFe LDH/GO	33.47	48.54	11.62	5.69	0.68
NiFe LDH/rGO	73.04	20	4.51	2.44	
NiMn LDH/rGO	66.55	23.58	8.82		1.05
NiMnFe LDH rGO	73.19	20.69	3.98	1.5	0.64

FT-IR, XRD Analysis

We compared FT-IR spectra of NiMnFe LDH, NiMnFe LDH/GO and NiMnFe LDH/rGO in order to clarify the differences (Figure S2). The GO characteristic bands at ~1600 and ~1700 cm^{-1} were assigned to nonoxygenated carbons (C=C) and carboxylic (-C=O) groups respectively, after the reduction process, most of the oxygenated functional groups vanished. In other words, these oxygenated groups almost disappeared in the spectra of NiMnFe LDH/rGO. However, C=C can be observed clearly in all rGO containing materials. These results demonstrated the successful fabrication and reduction of NiMnFe LDH/rGO nanohybrid.

What's more, XRD patterns of NiFe LDH, NiFe LDH/GO and NiFe LDH/GO were also contrasted (Figure S3). The strong intensity of peak at 2θ=11.513° was corresponding to the (003) plane of NiFe-LDH and the XRD pattern of peak (003) in NiFe LDH/GO and NiFe LDH/rGO was similar to that of NiFe-LDH, that may be there was only a small amount of rGO in the composites. However the diffraction peaks of (003) plane became weaker, which was because that the presence of GO and rGO brings down the crystallinity of the NiFe LDH/GO and NiFe LDH/rGO. And the diffraction peaks at smaller angles (i.e., (012)) of NiFe LDH/GO and NiFe LDH/rGO slightly shifted toward smaller angles compared with NiFe-LDH, these could be attributed to the incorporation of GO and rGO. The FWHM of (003) plane in NiFe LDH/GO and NiFe LDH/rGO was different.

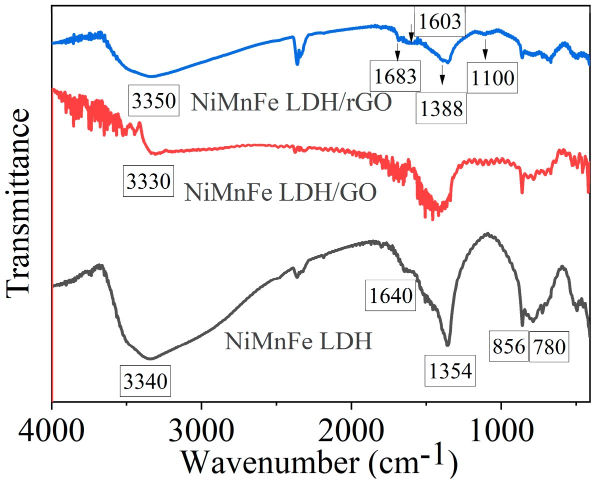

Figure S2. FT-IR spectra of NiMnFe LDH, NiMnFe LDH/GO and NiMnFe LDH/rGO.

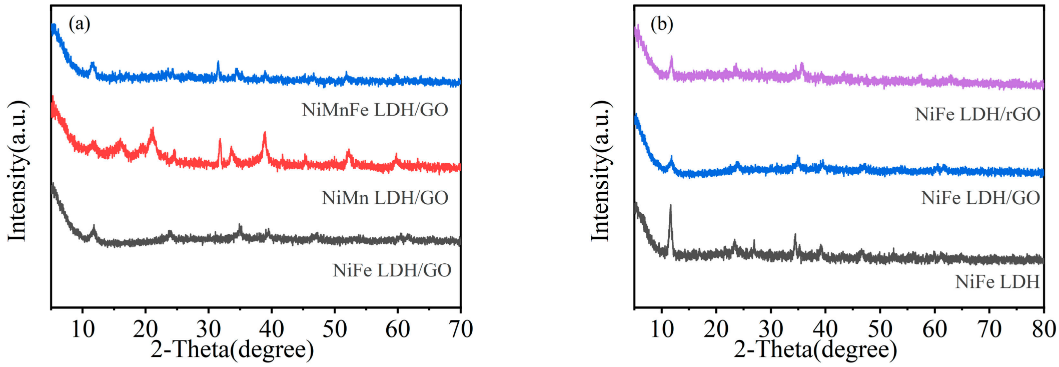

Figure S3. XRD patterns of (a) NiFe LDH/GO, NiMn LDH/GO, NiMnFe LDH/GO and (b) NiFe LDH, NiFe LDH/GO, NiFe LDH/rGO.

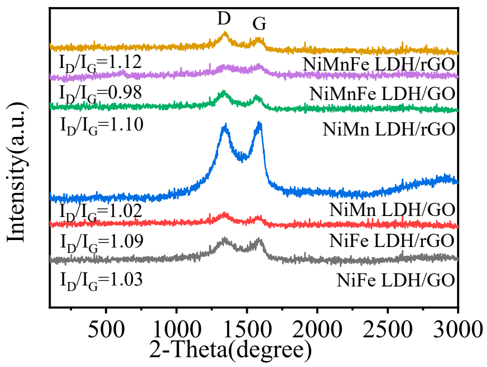

Figure S4. Raman spectra of NiFe LDH/GO, NiFe LDH/rGO, NiMn LDH/GO, NiMn LDH/rGO, NiMnFe LDH/GO, and NiMnFe LDH rGO.

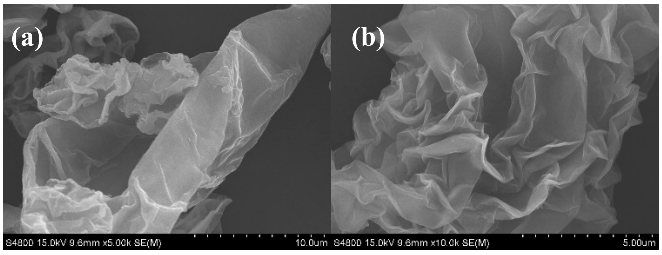

Figure S5. SEM images of GO. The scales for GO are 10 μm (a) and 5 μm (b), respectively.

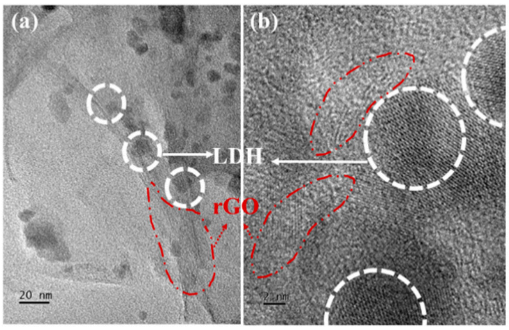

Figure S6. TEM image (a) and HRTEM image (b) of NiFe LDH/rGO.

Figure S7 showed the energy spectra of the three LDH/rGO catalysts, NiFe LDH/rGO, NiMn LDH/rGO and NiMnFe LDH/rGO. It can be found that the atomic percentage each metal element was nearly 1:1 or 1:1:1, which was consistent with the molar ratio of the raw material we added. And the weight ratio was about 20-30%.

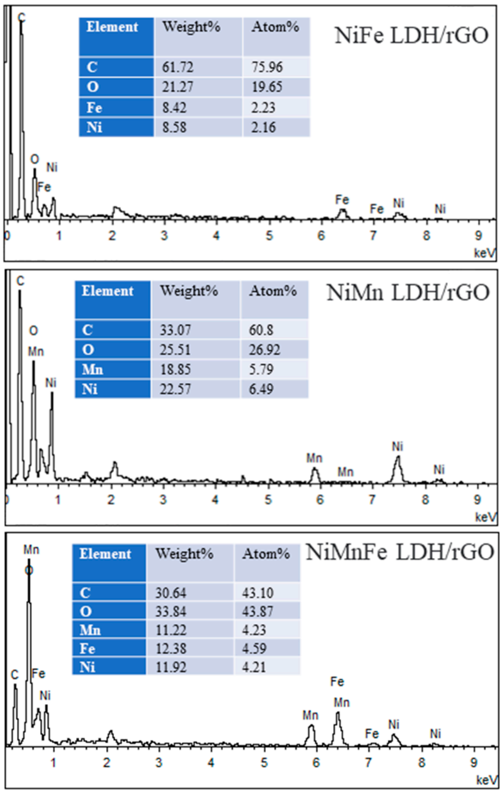

Figure S7. EDS spectra of different catalysts.

Specific surface area and pore size analysis

The specific surface area and pore size distribution of different catalysts were further tested by nitrogen adsorption/desorption isotherms (BET), as shown in Table S1 and Figure S8. All catalysts showed type IV isotherms with H3 hysteresis loops at relative pressures (P/P0) of 0.5~0.95, indicating the presence of a large number of mesopores. In addition, the inflection point in the N_2 desorption isotherm of NiFe LDH catalyst at 0.45~0.5 relative pressure indicates a significant increase in porosity. Table S1 shows that the BET specific surface area and pore volume of NiFe LDH are as high as 49.3 m^2g^{-1} and 0.19 cm^3g^{-1}, which is slightly higher than that of NiMnFe LDH (38.8 m^2g^{-1} and 0.19 cm^3g^{-1}), which is related to the larger spherical diameter of NiFe LDH catalysts, and the larger volume implies more pores, which is in agreement with the result of the morphology diagram. The specific surface area and pore volume of NiFe LDH and NiMnFe LDH were much higher than those of NiMnFe LDH (1.9 m^2g^{-1} and 0.005 cm^3g^{-1}), and the increase in specific surface area and pore volume was attributed to the loose spherical structure of the pores, whereas the latter was simply lamellar and stacked in different ways without forming more pores. The self-assembly of LDHs with rGO layers after stripping brings about the destruction of the original LDHs structure and reduces the pore space, and the composite with rGO increases the specific surface area, but it can be found that all the composite catalysts with LDHs/rGO have a smaller specific surface area than the corresponding LDHs. In addition, the loose spherical structure with many pores facilitates the generation of defects and oxygen vacancies. In general, a higher specific surface area exposes more active sites, and the mesoporous structure promotes the transport of reactants and actives, which is conducive to the activation of H_2O_2 to form a highly active oxidant.

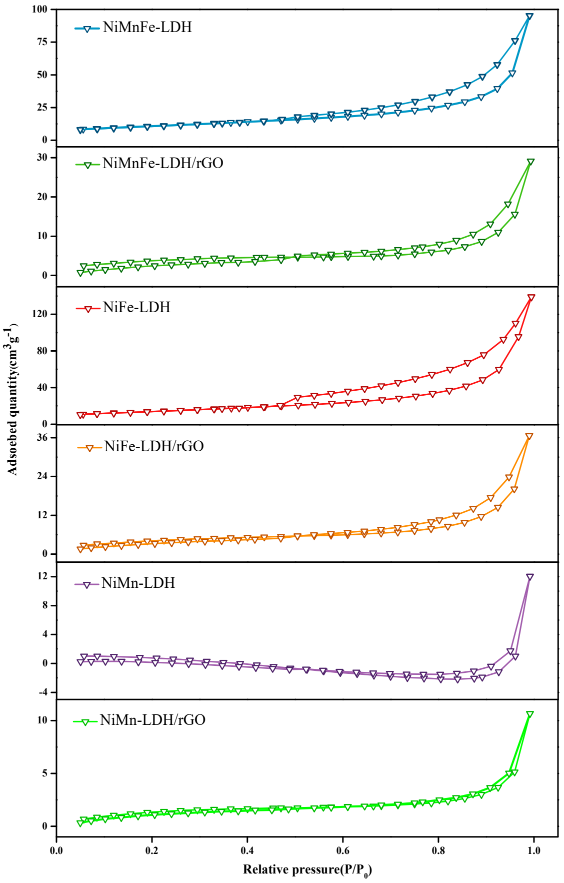

Figure S8. BET curves of NiFe LDH, NiMn LDH, NiMnFe LDH, NiFe LDH/rGO, NiMn LDH/rGO and NiMnFe LDH/rGO.

X-ray Photon Spectroscopy (XPS) Analysis

In order to obtain the elemental composition of the material and the valence distribution of each element, the samples were subjected to XPS testing. As shown in Figure S9 (a-c), for NiFe LDH, NiFe LDH/GO and NiFe LDH/rGO, the XPS full spectrum showed obvious C 1s, O 1s, Ni 2p, Fe 2p peaks, which indicated that C, O, Ni, Fe were the main elements constituting the three catalysts, and for NiMn LDH, NiMn LDH/GO and NiMn LDH/rGO, the XPS full spectrum showed obvious C 1s, O 1s, Ni 2p, Mn 2p peaks. For NiMn LDH, NiMn LDH/GO and NiMn LDH/rGO, there are obvious C 1s, O 1s, Ni 2p and Mn 2p peaks in the full spectrum of XPS, and NiMnFe LDH and NiMnFe are composed of the major elements of C, O, Ni, Mn and Fe. The peak intensities of these catalysts are all larger, indicating that the preparation is more effective.

The proportions of all the key elements in LDHs/GO and LDHs/rGO were listed in Table S3. Compared with LDHs/GO, it was obvious to observe that the content of element C in their corelated LDHs/rGO was sharply increased (from about 40 at% to about 70 at%), whereas the O content was significantly decreased(from about 40 at% to about 20 at%), manifesting that GO in the NiFe LDH/GO, NiMn LDH/GO and NiMnFe LDH/GO was partially reduced to generate NiFe LDH/rGO, NiMn LDH/rGO and NiMnFe LDH rGO.

High-resolution XPS spectra Figure S9 (d) Ni 2p region consists of Ni 2p1/2 and Ni 2p3/2 and two satellite peaks. the Ni 2p1/2 and Ni 2p3/2 peaks can be further divided into four peaks of 874.5, 873.5, 857.0 and 856.2 eV. the two peaks with lower binding energies (873.5 and 856.2 eV) correspond to Ni^{2+}, while the two peaks with binding energies of 874.5 and 857.0 eV are characteristic peaks of Ni^{3+}. The two peaks with lower binding energies (873.5 and 856.2 eV) correspond to Ni^{2+}, while the two peaks with binding energies of 874.5 and 857.0 eV are characteristic of Ni^{3+}.

XPS Fe 2p spectra Figure S9 (e) shows that the Fe 2p1/2 and Fe 2p3/2 peaks can be categorized into a pair of peaks at 713.5 eV and 725.7 eV attributed to Fe^{3+} species, and a pair of peaks at 710.6 eV and 723.4 eV attributed to Fe^{2+} species with

two satellite peaks (716.2 eV and 728.9 eV).

The Mn 2p spectra are shown in Figure S9 (f), from which it can be seen that there are two spin orbitals in Mn 2p, and two characteristic peaks appear at 641.4 eV and 652.9 eV, which correspond to Mn 2p3/2 and Mn 2p1/2, respectively, and the two peaks can be divided into four peaks after peak splitting into 4 peaks as 640.8 and 651.6 eV for the Mn^{2+} characteristic peaks, 643.2 and 654.5 eV for Mn^{3+} characteristic peaks, and another pair of satellite peaks as 646.7 and 658.0 eV. 654.5 eV are the characteristic peaks of Mn^{3+}, and there are a pair of satellite peaks of 646.7 and 658.0 eV, which suggests that Mn is mainly present in NiMn LDH and NiMn LDH/rGO at +2 valence and +3 valence.

This suggests that mixed-valence redox pairs of Fe^{2+}/Fe^{3+} and/or Ni^{2+}/Ni^{3+} and/or Mn^{2+}/Mn^{3+} coexist in these catalysts, NiFe LDH, NiMn LDH, NiMnFe LDH, NiFe LDH/rGO, NiMn LDH/rGO, and NiMnFe LDH/rGO, and that electron transfer between these mixed-valence redox pairs can activate the in situ generation of ·OH at the cathode for the degradation of target pollutants. The electron transfer between these mixed-valence redox pairs can activate the in situ generation of ·OH from H_2O_2 at the cathode for the degradation of target pollutants.

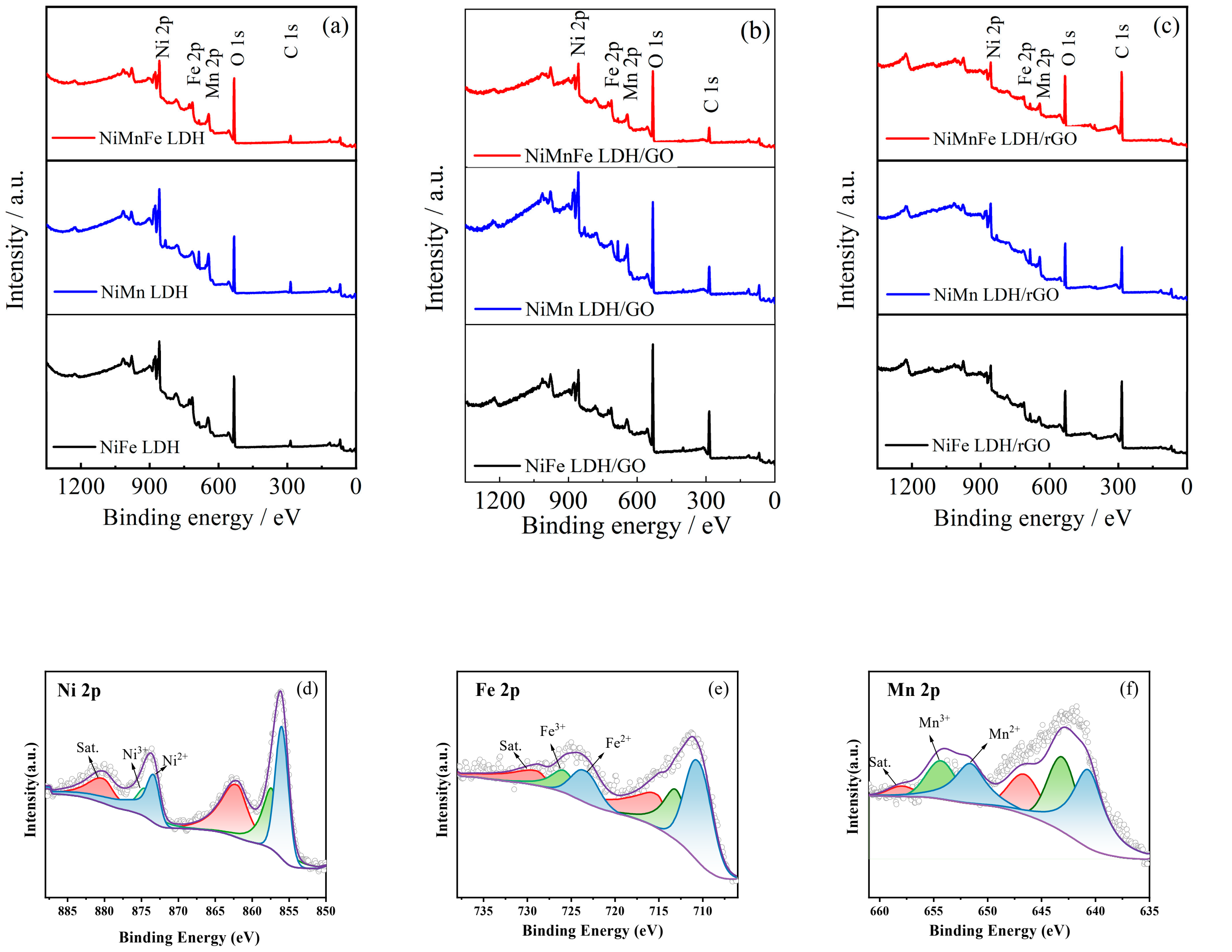

Figure S9. Wide XPS spectra of (a) NiFe LDH, NiMn LDH, NiMnFe LDH catalysts, (b) NiFe LDH/GO, NiMn LDH/GO, NiMnFe LDH/GO catalysts, (c) NiFe LDH/rGO, NiMn LDH/rGO, NiMnFe LDH/rGO catalysts, and high-resolution scanning XPS spectra for (d) Ni 2p, (e) Fe 2p of NiFe LDH/rGO, (f) Mn 2p of NiMnFe LDH/rGO.

Electrochemical Impedance Spectroscopy (EIS) Analysis

To further evaluate the electron transfer ability of the catalysts, the catalytic performance of the modified electrodes in a mixed solution of $K_3[Fe(CN)_6]$ and KCl was investigated by using the EIS characterization method. At a certain voltage, the redox reaction in the solution occurs gradually, and the reacting ions are potassium ferrocyanide ions and potassium ferrocyanide ions, so the EIS curve in potassium ferrocyanide solution can be used to examine the electrochemical properties of the material. Figure S10 shows the Nyquist curves of three composite electrodes, NiFe LDH/rGO, NiMn LDH/rGO and NiMnFe LDH/rGO, in a mixed solution of $K_3[Fe(CN)_6]$ (5 mM) and KCl (0.1 M). As can be seen from the figure, the curves of these three modified electrodes consist of a high-frequency region and a low-frequency region, with the high-frequency region having a semicircular arc curve and the low-frequency region resembling a straight line. The high-frequency part shows the resistance that exists between the reference electrode and the modified electrode, which is indicated by the intercept between the arc and the real axis; the diameter of the semicircle indicates the magnitude of the resistance of the modified electrode during the electron transfer process. Compared with the NiMn LDH/rGO and NiMnFe LDH/rGO modified electrodes, the NiFe LDH/rGO composite modified electrode shows a smaller radius of curvature in the high-frequency region, which indicates that the composite accelerates the electron transfer between the two ionic pairs mentioned above and their and the electrodes, with a lower charge-transfer resistance, and has a faster interfacial electron-transfer capability, thus promoting the Fe(III)/Fe (II) reduction process. In addition, the impedance of these three composite catalysts was in the range of 80-100 Ω, which may be due to the incorporation of rGO to improve the electrical conductivity of the composite catalysts, resulting in a decrease in the resistance during the electron transfer process, and therefore enhancing their electrochemical activity.

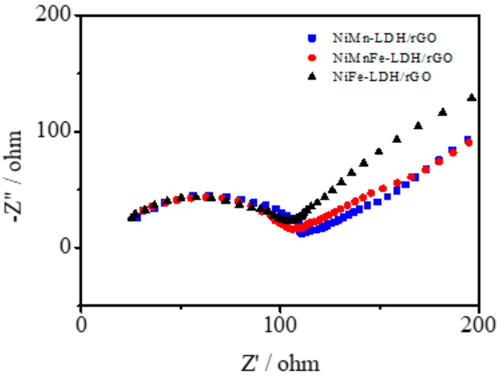

Figure S10. EIS plots of the electrocatalysts of NiFe LDH/rGO, NiMn LDH/rGO and NiMnFe LDH/rGO.

References

1. Xu X, Li Y, Zhang G, Yang F, He P. Nio-$NiFe_2O_4$-rGO Magnetic nanomaterials for activated peroxymonosulfate degradation of rhodamine B [J]. *Water,* **2019**, *11*(2): 384

2. Liu Y, Li Z, Chen W, Feng X. Fast determination of rutin on a biosensor made using a layered double hydroxide nanocomposite modified electrode [J]. Biosensors, **2024**, 14(1): 18.

3. Xu J, Tang R, Liu M, Xie S, Zhang D, Kong X, Jin S, Ji H, Zhang T. Enhancing the catalytic activity of layered double hydroxide supported on graphene for lithium-sulfur redox reactions[J]. *Batteries,* **2022**, 8(11): 200.

4. Álvarez MG, Crivoi DG, Medina F, Tichit D. Synthesis of chalcone using LDH/graphene nanocatalysts of different compositions [J]. *ChemEngineering,* **2019**, 3(1): 29.
